# Editorial: Cognitive inspired aspects of robot learning

**DOI:** 10.3389/fnbot.2023.1256788

**Published:** 2023-08-24

**Authors:** Francisco Cruz, Miguel A. Solis, Nicolás Navarro-Guerrero

**Affiliations:** ^1^School of Computer Science and Engineering, University of New South Wales, Sydney, NSW, Australia; ^2^Escuela de Ingeniería, Universidad Central de Chile, Santiago, Chile; ^3^Facultad de Ingenieria, Universidad Andres Bello, Santiago, Chile; ^4^L3S Research Center, Leibniz Universität Hannover, Hannover, Germany

**Keywords:** robotics, cognitive robotics, robot learning and behavior adaptation, bio-inspired robotics, social robotics/HRI engineers

Robot learning enables robots to acquire new knowledge and skills through experience and interaction with their environment. Robot learning involves developing algorithms that allow robots to learn autonomously, adapt to new situations, and improve their performance over time. Using machine learning, robots can analyze large amounts of data and extract patterns to make decisions. This approach allows robots to learn from past experiences and apply that knowledge to future tasks, ultimately enhancing their capabilities and versatility.

However, although machine learning has shown great potential in robot learning, it also faces several challenges and limitations. One significant problem, for instance, is the issue of data scarcity. Collecting sufficient and diverse data for training robots can be complex and time-consuming (Navarro-Guerrero et al., 2023). Unlike traditional machine learning applications where large datasets might be available, gathering data for robot learning often requires physical interactions and real-world environments, which can be expensive and challenging (Navarro-Guerrero et al., 2023).

Cognitive robotics combines principles from cognitive science and robotics and offers some advantages for robot learning and intelligence. For instance, cognitive robotics offers the potential to create intelligent and interactive robots (Bignold et al., 2021) that can understand, reason, learn, and adapt like living organisms (Navarro-Guerrero et al., 2017). Combining cognitive science and robotics insights allows robots to overcome limitations associated with traditional robotic systems and machine learning methods (Millan-Arias et al., 2021). Therefore, Cognitive Robotics is a paradigm for developing more sophisticated and versatile machines capable of operating in complex real-world environments.

According to Krichmar (2018), there is a close relationship between neurorobotics and cognitive robotics. Krichmar suggests that the community should focus more on general cognition rather than just specific brain areas or behaviors. He also emphasizes the importance of coupling the brain, body, and environment and studying the brain in the context of naturalistic behaviors.

In this Research Topic, we aimed at novel contributions to new theoretical methods and applications in cognitively inspired aspects of robot learning, such as:

- Development of skills in biological systems and robots.
- Principles and theories of development and learning.
- Self-organizing behavior.
- Models of human-human and human-robot interaction.
- Human-in-the-loop reinforcement learning and applications.
- Multi-robot systems with human collaboration.
- Non-verbal and multimodal interaction.
- Models on active learning.
- Architectures for lifelong learning.
- The emergence of body and affordance perception.
- Bio-inspired perceptual systems.
- Continual sensorimotor learning.
- Models for prediction, planning, and problem-solving.
- Ethics and trust in computational intelligence and robotics.
- Ethical reasoning and moral uncertainty.
- Social learning in humans, animals, and robots.
- Explainable robotic systems.
- Robot prototyping of human and animal skills.
- Models of perception in biological and artificial agents.
- Cognitive computing.

In separate paragraphs, we describe the six accepted contributions, highlighting their novelty and significance in addressing some cognitive-inspired robot learning challenges.

The first paper by Li et al. introduced a novel approach to robotic grasping using a neuromorphic vision sensor. The authors focused on the challenge of detecting robotic grasps in a dynamic camera view of a scene with various objects. To tackle this task, they built a large-scale and well-annotated robotic grasping dataset called Event-Grasping, which consisted of 91 objects. In addition, this work proposed a spatial-temporal mixed particle filter to track the LED-based grasp rectangles and a deep neural network architecture for grasp detection. Notably, they approached the angle learning problem as a classification task rather than a regression. The results of their method demonstrated a significantly high accuracy when evaluated on the proposed Event-Grasping dataset.

Cong et al. presented a vision-proprioception model explicitly designed for planar object-pushing tasks. Their model efficiently integrated all the necessary environmental information to enable successful pushing actions. To extract task-relevant information from the visual input, they employed a variational autoencoder to generate compact image representations. These representations were combined with real-time robot state data from the hardware system, including the robot's end-effector position to form the state representation for a Markov Decision Process. They used the Soft Actor-Critic algorithm to train the robot to push objects to target positions within a simulation environment. This algorithm enabled the robot to learn optimal pushing policies from various random initial object poses. Through their experiments, the authors demonstrated the effectiveness of their proposed algorithm. It outperformed a state-based baseline model and surpassed existing policies that worked directly on raw image observations. The results showcased superior pushing performance achieved by their vision-proprioception model.

Abel et al. conducted a study focusing on how individuals perceive robots and their actions. The researchers utilized functional magnetic resonance imaging (fMRI) to analyze brain activations of both female and male participants while observing movements performed by either a human or a robot model, which were rated for their anthropomorphic and robotic likeness. The findings revealed that observing the human model and anthropomorphic movements led to similar activations in the posterior temporal and parietal areas associated with biological motion coding. On the other hand, observing the robot model predominantly activated areas within the ventral stream, while robotic movements primarily engaged the primary and higher-order motor areas. Notably, this latter activation was primarily observed in female participants, while male participants displayed activations in the posterior parietal cortex for both the robot model and robotic movements. The overall analysis indicated that men rely on the ventro-dorsal stream, utilizing previous knowledge to analyze movements. In contrast, female participants employed the dorso-dorsal and ventral streams to analyze movement differences and distinguish between different models.

Han et al. proposed a novel approach for addressing multiple semantic manipulation instructions using semantic-goal-conditioned reinforcement learning. The authors introduced a conservative curiosity-driven method called mutual information motivation with a hybrid policy mechanism (MIHM) to address challenges related to uncontrollability and distraction. MIHM encompassed two main contributions. Firstly, it incorporated decoupled mutual information-based intrinsic motivation, which prevented the agent from being driven to explore potentially dangerous states due to uncontrollable curiosity. Secondly, it featured a precisely trained and automatically switched hybrid policy mechanism, effectively eliminating distractions from the curiosity-driven policy and enabling optimal utilization of exploration and exploitation. In a sparse-reward robotic manipulation task, MIHM demonstrated the fastest learning speed compared to four state-of-the-art curiosity-driven methods. Importantly, MIHM was the only approach among the baselines to stack three objects successfully.

Abpeikar et al. conducted a study on achieving swarming collective motion in groups of mobile robots through an iterative transfer learning approach. The authors employed transfer learning to enable a deep learning model to recognize and adjust stable collective motion behaviors across multiple robot platforms. The transfer learner approach required only a small set of initial training data collected from the random movements of each robot. The proposed approach iteratively updated its knowledge base, eliminating the need for extensive data collection and avoiding the risks associated with trial-and-error learning on physical robot hardware. The method's effectiveness was tested on two robot platforms: simulated Pioneer 3DX robots and real Sphero BOLT robots. The transfer learning approach enabled both platforms to adjust automatically and exhibit stable collective behaviors. Furthermore, by utilizing the knowledge-base library, the tuning process was both fast and accurate, enhancing the overall efficiency of the approach.

Hafez et al. focused on addressing the issue of catastrophic forgetting in deep reinforcement learning agents. They proposed a novel cognitive-inspired replay memory approach based on the Grow-When-Required self-organizing network, which resembles a map-based mental model of the world. The approach organized stored transitions into a compact network. Similar samples were merged to reduce memory size while increasing the pair-wise distance among samples. This consolidation process enhanced the relevancy of each sample within the memory. The study demonstrated that adopting a map-based experience replay technique significantly reduced memory usage while only resulting in minor performance decreases. This approach provided a promising solution to mitigate catastrophic forgetting in deep reinforcement learning agents.

The guest editors greatly appreciate the patience, diligence and dedicated efforts of all the reviewers, many of whom made insightful recommendations that helped push the quality of some of the submissions to well beyond the recommended threshold for acceptance into the Research Topic. We would also like to thank the Specialty Chief Editors of the Frontiers in Neurorobotics journal, Alois C. Knoll and Florian Röhrbein, for the opportunity to run this Research Topic, as well as the editorial staff, Fiona Robertson, Valeria Corlianò, Marta Benedekova, Hannah Mellor, Naomi Wawa, Marina Solares, and Oliver Boylan for their assistance during the collation of articles and processing of this Research Topic. Finally, The guest editors also thank all the authors, especially for their valuable contribution to the journal and this Research Topic.

## Author contributions

FC: Conceptualization, Writing—original draft, Writing—review and editing. NN-G: Conceptualization, Writing—original draft, Writing—review and editing. MS: Conceptualization, Writing—original draft, Writing—review and editing.
